# Editorial: Advances in Plant Meiosis: From Model Species to Crops

**DOI:** 10.3389/fpls.2019.01627

**Published:** 2019-12-18

**Authors:** Tomás Naranjo, Changbin Chen, Zhukuan Cheng, Mónica Pradillo

**Affiliations:** ^1^ Departamento de Genética, Fisiología y Microbiología, Facultad de Biología, Universidad Complutense de Madrid, Madrid, Spain; ^2^ Department of Horticultural Science, University of Minnesota, St. Paul, MN, United States; ^3^ State Key Laboratory of Plant Genomics and Center for Plant Gene Research, Institute of Genetics and Developmental Biology, Chinese Academy of Sciences, Beijing, China

**Keywords:** chromosome sorting, chromatin organization, telomere dynamics, double-strand breaks, crossovers, homoeologous recombination, *Ph1*

Advances in the study of plant meiosis produced in the last three decades were based mainly in the isolation of meiotic mutants and genes in the model species *Arabidopsis thaliana* and in other species such as rice or maize. Chromosome mutants produced in wheat provided also valuable information. Nowadays, many research groups are using translational biology approaches to improve the sustainability of food production in crops based on the manipulation of meiotic recombination. Because many crops are polyploids, complications of the meiotic behavior derived from the polyploid condition are also in the focus of a number of research projects. The purpose of this Research Topic was to provide a platform for the publication of updated information and high-quality research papers, in both model species and crops, which will represent a guide addressing our fundamental knowledge of meiosis as well as its implications for plant breeding. After a large response, which reflects active research being undertaken, we published a total of 21 papers, including 1 Mini-Review, 3 Reviews, 1 Methods, and 16 Original Research. These papers are grouped in the following items: i) new approaches and methods in the study of meiosis; ii) meiotic recombination; iii) meiosis in polyploids; iv) chromosome segregation; and v) other aspects of meiosis.

i) New approaches and methods in the study of meiosis presents one review paper and four technological articles outlining recent advancement of technologies for plant meiosis studies ranging from genomic analysis, single cell sequencing to super-resolution and 3D imaging investigation of chromatin dynamics. All tools available for studying meiosis have been reviewed comprehensively by Lambing and Heckmann, which include super-resolution microscopy and live cell imaging that are extensively used in understanding chromosome dynamics and plant meiosis progression, application of sequencing-based techniques to map crossovers (COs) and their correlation to gene expression landscapes, as well as the proteomic analysis to profile proteins involved in meiosis. Capilla-Perez et al. report a recent development of genetic resources by creating a new homozygous mutant library for researchers to perform functional analysis of meiotic genes. Eight hundred ninety-seven homozygous *Arabidopsis* mutant lines are among this collection, including those targeted 26 previously reported meiotic genes. Dreissig et al. use single pollen nucleus sequencing to study meiotic recombination events and segregation distortion caused by postmeiotic processes. Genome wide distribution of recombination uncovered similarity of recombination landscapes between barley pollen and double haploid plants; but the segregation distortion is high in double haploid population comparing to almost absent in pollen. Sepsi et al. combine immunolabeling, fluorescence *in situ* hybridization, and confocal microscopy to simultaneously detect the nuclear localization of proteins and specific DNA sequences within chromosomes. Construction of 3D images has shown detailed chromosome structures and critical proteins associated with meiosis in rye. Also using rye system, Hesse et al. report a technique combining scanning electronic microscopy and fluorescence microscopy to examine ultrastructure and dynamics of synaptonemal complex (SC) components during meiotic pairing and synapsis. The super high-resolution technique advances the ability of researchers to study chromosome dynamics and configuration during meiosis with great details at the molecular and subcellular levels.

ii) Meiotic recombination is initiated by the formation of DNA double-strand breaks (DSBs). A subset of these DSBs are repaired as COs, reciprocal exchanges of genetic information between homologous chromosomes, allowing the formation of genetically unique gametes. COs are non-randomly distributed along chromosomes, there are regions that recombine more than average (hotspots), while other regions are completely devoid of COs. Okagaki et al. compile historically accumulated evidence to explain that in maize, unlike the situation in other model systems, many recombination hotspots are located within genes. In some of these genes there is a gradient of recombination (polarity) with higher CO rates at the ends (5′ or 3′). As well as in *Arabidopsis*, in maize, the fraction of DSB hotspots resolving as COs is small (less than 4%). The message from this detailed review is that the recombination process of a species does not necessarily fit to those of the model species described. In addition, it is necessary to compile data derived from different methodological approaches in order to have a global view of how the meiotic recombination process takes place. Meiotic recombination is a process highly regulated by specific genes. Among these genes are those involved in processing DSBs. The work by Wang et al. describes the consequences arising from the absence of ZmCOM1 during maize meiosis. They demonstrate that this protein is indispensable to ensure the bouquet formation and the assembly of the SC. In addition, maize plants deficient for this protein display problems during mitosis. According to their observations, the function of COM1 is conserved in plant meiosis. However, during the cell cycle, its function seems to be more important in maize than in plants with smaller genomes. Hu et al. analyze the role of the replication factor C–like protein OsRAD17. This protein, together with the 9-1-1 complex, is essential for DSB repair during rice meiosis, and collaborates with the meiosis-specific ZMM proteins to ensure correct homologous pairing and synapsis. In plant crops, many genes that contribute to agronomically important traits are located within CO suppressed regions. One of the factors that regulate CO frequency is the anti-CO protein Fanconi Anemia Complementation Group M (FANCM). The absence of this protein produces a 3-fold increase in CO formation in *Arabidopsis*, although this increase is not uniform along chromosomes. Blary et al. demonstrate that the anti-CO function of FANCM is also conserved in the crop species *Brassica rapa* and *B. napus*, highlighting the potential application of this gene in plant breeding programs. Zelkowski et al. prove that the meiotic function of the SMC5/6 complex that has been demonstrated in yeasts, worms, and mammals is conserved in plants. Specifically, one δ-kleisin subunit of this complex, AtNSE4A, is essential for the resolution of meiotic recombination intermediates during the first meiotic division in *Arabidopsis*. Furthermore, during prophase I, the NSE4A protein colocalizes with the central element of the SC (ZYP1) in *Arabidopsis*, *B. rapa*, and rye, revealing also a possible role in synapsis.

iii) Meiosis in polyploids includes papers concerning the analysis of preferences on interactions between homologous and identical chromosomes in autotreploids, the identification and mode of action of the wheat *Ph1* gene, the study of the meiotic behavior of alien chromosomes added to wheat, and the identification of homoeologous pairing between wheat and *Elytrigia elonagata* chromosomes. Parra-Núñez et al. report the formation of more multivalents than expected under the assumption of simple random-end pairing in autotetraploids of two accessions of *Arabidopsis*. This suggests more than two autonomous synapsis initiation sites per chromosome and more than one partner switches per tetrasome. The multivalent frequency decreases in the tetraploid obtained after duplication of the hybrid between the two ecotypes, probably because of heterozygosity. Preferences for chiasma formation between homologous versus identical chromosomes in tetrasome 3 but not in tetrasome 2 of the duplicated intraspecific hybrid, reveal the existence of chromosome-specific mechanisms affecting the partner selection. Rey et al. use two TILLING mutants and one CRISPR mutant of the *TaZIP4-B2* gene on chromosome 5B to produce interspecific wheat × *Aegilops variabilis* hybrids whose meiotic phenotype identifies *ZIP4* as the *Ph1* gene. The frequency of interspecific homoeologous recombination induced by the *ZIP4* mutations increases after irrigation of the plants with magnesium of a nutrient solution. Naranjo argues that the meiotic behavior of individual rye chromosomes changes when they are introgressed into a wheat background. The pattern of chromatin organization at early prophase I is affected, especially for chromosome 4R, which increases its length much more than any other rye chromosome at leptotene-zygotene. Telomeres clustering, but not their dispersion, is depending of the chromosome conformation, which has implications on synapsis and recombination. Chiasma formation is affected in some chromosome arms that show complete synapsis. Calderón et al. use addition lines of barley and *Hordeum chilense* chromosomes into wheat to produce double monosomic additions carrying pairs of homoeologous or non-homoeologous *Hordeum* chromosomes. In the presence of *Ph1,* only the *Hordeum* homoeologous pairs recognize each other in subtelomeric regions and complete synapsis. However, they do not form chiasmata suggesting that *Ph1* suppresses homoeologous CO formation. He et al. use genomic *in situ* hybridization to identify the chromosomes of hexaploid wheat and decaploid *E. elongata* in interspecific hybrids and backcrosses of the hybrids with wheat. Some associations at metaphase I between chromosomes of both species are produced supporting the occurrence of interspecific homoeologous recombination. This agrees the hypothesis that *Ph-*suppressor genes, which promote homoeologous recombination, are present in *E. elongata*.

iv) Chromosome segregation includes three papers focusing on both chromosomal properties and environmental factors that impact on chromosome segregation. Easterling et al. use 3D cytogenetic analysis of meiotic chromosome dynamics in hop (*Humulus lupulus*) pollen mother cells. The configuration of chromosomes in hop male meiosis include multiple, atypical, non-disomic chromosome complexes detected as aneuploidy, segmental aneuploidy, or chromosome rearrangements, which implicate multiple contributing factors to segregation distortion in hop. Through analysis of multiple centromere misdivision derivatives of a translocation between the supernumerary B chromosome and the short arm of chromosome 9 in maize, Han et al. report that the property size of a centromere does not dramatically affect its segregation or its ability to progress to the poles at the end of cell division. The biochemical features of centromeres are likely the factors for the adjustment in response to the cellular conditions. Sudden temperature changes often lead to abnormal chromosome segregation. Liu et al. use the model system *A. thaliana* to explore the mechanisms behind the cold-induced male meiotic restitution. Findings from this research implicate that GA-DELLA signaling is not the critical factor responsible for the cold-induced abnormal chromosome segregation.

v) Other aspects of meiosis include three reviews that individually focus on the influences of exogenous anthropogenic factors in plant meiosis, the effects of recombination regulation on speciation success of polyploidy species, and the technical approaches to engineer key meiotic genes in tropical crops. Plants now grow on environments that are frequently exposed to anthropogenic factors capable of modulating their meiotic processes. In the first review, Fuchs et al. discuss major anthropogenic factors affecting meiosis in plants, including environmental stresses, agricultural inputs, heavy metals, pharmaceuticals and pathogens. In most cases, the anthropogenic impacts on meiosis are altered recombination frequency and distribution, severe chromosomal fragments caused by stickiness and bridges, precocious movements, and unequal separations resulting in micronuclei and aneuploidy, as well as spindle aberrations. Polyploid speciation is closely related to the regulation of meiotic recombination, which is reviewed by Pelé et al. To achieve speciation success of a polyploidy species, three aspects of meiotic recombination should be guaranteed: the regulation of the genetic variability of newly formed polyploids, the maintenance of the allelic combinatorial possibilities in the following generations, and the faithful segregation of multiple homologs or homoeologs in auto- or allopolyploids. During speciation, different patterns of recombination, coupled with different polyploid formation pathways, cause varied level of heterozygosity. The formation of a polyploidy species with high level of heterozygosity will produce more genetically diverse progenies, therefore improving its environmental adaptability through different natural selections. In the third review, Bolaños-Villegas and Argüello-Miranda mainly summarized the possible technical approaches to manipulate meiosis in orphan crop breeding. They proposed that genome haploidization through modified CENH3 or the apomixes-inducing genes could be a rapid option to create new crosses in tropical crops. Moreover, gene editing of some key meiotic genes, such as *ASY1*, *FANCM*, *RECQ4*, and *PH1*, might facilitate produce new varieties that are enriched in desirable wild traits.

## Author Contributions

TN, CC, ZC, and MP wrote the paper.

## Funding

Work in the laboratory of TN and MP was supported by grant AGL2015-67349-P from Dirección General de Investigación Científica y Técnica, Ministerio de Economía y Competitividad of Spain. Research in the laboratory of CC is sponsored by the National Science Foundation (NSF-IOS-1546792).

## Conflict of Interest

The authors declare that the research was conducted in the absence of any commercial or financial relationships that could be construed as a potential conflict of interest.

